# Genetic manipulation of insulin/insulin-like growth factor signaling pathway activity has sex-biased effects on *Drosophila* body size

**DOI:** 10.1093/g3journal/jkaa067

**Published:** 2021-04-01

**Authors:** Jason W Millington, George P Brownrigg, Paige J Basner-Collins, Ziwei Sun, Elizabeth J Rideout

**Affiliations:** Department of Cellular and Physiological Sciences, Life Sciences Institute, The University of British Columbia, Vancouver, BC V6T 1Z3, Canada

**Keywords:** *Drosophila*, sex, insulin pathway, body size, genetics

## Abstract

In *Drosophila* raised in nutrient-rich conditions, female body size is approximately 30% larger than male body size due to an increased rate of growth and differential weight loss during the larval period. While the mechanisms that control this sex difference in body size remain incompletely understood, recent studies suggest that the insulin/insulin-like growth factor signaling pathway (IIS) plays a role in the sex-specific regulation of processes that influence body size during development. In larvae, IIS activity differs between the sexes, and there is evidence of sex-specific regulation of IIS ligands. Yet, we lack knowledge of how changes to IIS activity impact body size in each sex, as the majority of studies on IIS and body size use single- or mixed-sex groups of larvae and/or adult flies. The goal of our current study was to clarify the body size requirement for IIS activity in each sex. To achieve this goal, we used established genetic approaches to enhance, or inhibit, IIS activity, and quantified pupal size in males and females. Overall, genotypes that inhibited IIS activity caused a female-biased decrease in body size, whereas genotypes that augmented IIS activity caused a male-specific increase in body size. These data extend our current understanding of body size regulation by showing that most changes to IIS pathway activity have sex-biased effects, and highlights the importance of analyzing body size data according to sex.

## Introduction

Over the past two decades, the *Drosophila* larva has emerged as an important model to study the molecular and developmental processes that contribute to final body size. When nutrients are plentiful, one important factor that affects body size in most *Drosophila* species is whether the animal is male or female: female flies are typically larger than male flies (Alpatov 1930; Pitnick *et al.*1995; French *et al.* 1998; Huey *et al.* 2006; Okamoto *et al.* 2013; Testa *et al.* 2013; Rideout *et al.* 2015; Sawala and Gould 2017; reviewed in Millington and Rideout 2018). This increased body size is due to an increased rate of larval growth and sexually dimorphic weight loss in wandering larvae, as the duration of the larval growth period does not differ between the sexes in wild-type flies (Okamoto *et al.* 2013; Testa *et al.* 2013; Sawala and Gould 2017). While the precise molecular mechanisms underlying the male–female difference in body size remain incompletely understood, recent studies have revealed a key role for the insulin/insulin-like growth factor signaling pathway (IIS) in the sex-specific regulation of developmental processes that influence body size (Shingleton *et al.* 2005; Grönke *et al.* 2010; Testa *et al.* 2013; Rideout *et al.* 2015; Liao *et al.* 2020; Millington *et al.* 2021).

Normally, IIS activity is higher in female larvae than in age-matched males (Rideout *et al.* 2015; Millington *et al.* 2021). Given that increased IIS activity is known to promote cell, tissue, and organismal size (Grewal 2009; Teleman, 2010), this suggests that elevated IIS activity is one reason that females have a larger body size. Indeed, the sex difference in body size was abolished between male and female flies carrying a mutation that strongly reduced IIS activity (Testa *et al.* 2013), and between male and female pupae reared on diets that markedly decrease IIS activity (Rideout *et al.* 2015). In both cases, the sex difference in body size was eliminated by a female-biased decrease in body size (Testa *et al.* 2013; Rideout *et al.* 2015). While these findings suggest that IIS plays a role in sex-specific body size regulation during development, only one genetic combination was used to reduce IIS activity (Testa *et al.* 2013). Therefore, it remains unclear whether the sex-biased effect of reduced IIS activity on body size is a common feature of genotypes that alter IIS activity.

In the present study, we used multiple genetic approaches to either enhance or inhibit IIS activity, and monitored body size in males and females. While previous studies show that the genetic approaches we employed effectively alter IIS activity, the body size effects in each sex remain unclear due to frequent use of mixed- or single-sex experimental groups, and the fact that statistical tests to detect sex-by-genotype interactions were not applied (Fernandez *et al.* 1995; Chen *et al.* 1996; Leevers *et al.* 1996; Böhni *et al.* 1999; Brogiolo *et al.* 2001; Cho *et al.* 2001; Rintelen *et al.* 2001; Britton *et al.* 2002; Ikeya *et al.* 2002; Rulifson *et al.* 2002; Géminard *et al.* 2009; Zhang *et al.* 2009; Grönke *et al.* 2010). Our systematic examination of IIS revealed most genetic manipulations that reduced IIS activity caused a female-biased reduction in body size. In contrast, most genetic manipulations that enhanced IIS activity increased male body size with no effect in females. Together, these findings provide additional genetic support for IIS as one pathway that impacts sex-specific body size regulation in *Drosophila*.

## Materials and methods

### Fly husbandry

*Drosophila* growth medium consisted of: 20.5 g/L sucrose, 70.9 g/L D-glucose, 48.5 g/L cornmeal, 45.3 g/L yeast, 4.55 g/L agar, 0.5 g CaCl_2_•2H_2_O, 0.5 g MgSO_4_•7H_2_O, 11.77 mL acid mix (propionic acid/phosphoric acid). Diet data were deposited under “Rideout Lab 2Y diet” in the *Drosophila* Dietary Composition Calculator (Lesperance and Broderick 2020). Larvae were raised at a density of 50 animals per 10 mL food at 25°C, and sexed by gonad size. Adult flies were maintained at a density of 20 flies per vial in single-sex groups.

### Fly strains

The following fly strains from the Bloomington *Drosophila* Stock Center were used: *w^1118^* (#3605), *UAS-rpr* (#5823), *UAS-Imp-L2-RNAi* (#55855), *InR^E19^* (#9646), *InR^PZ^* (#11661), *Df(3R)Pi3K92E^A^* (#25900), *chico^1^* (#10738), *foxo^21^* (#80943), *foxo^25^* (#80944), *r4-GAL4* (fat body), and *dilp2-GAL4* (insulin-producing cells [IPCs]). Additional fly strains include: *UAS-Kir2.1* (Baines *et al.* 2001), *dilp1*, *dilp3, dilp4, dilp5, dilp6^41^, dilp7, Df(3L)ilp2-3,5, Df(3L)ilp1-4,5* (Grönke *et al.* 2010)*, Sdr^1^* (Okamoto *et al.* 2013), *Pi3K92E^2H1^* (Halfar *et al.* 2001), *Pdk1^4^* (Rintelen *et al.* 2001), *Akt1^3^* (Stocker *et al.* 2002)*.* All fly strains except *dilp6^41^* were backcrossed into a *w^1118^* background for 6 generations. All strains without a visible marker were crossed six times to a *w^1118^* strain carrying a balancer chromosome corresponding to the genomic location of the gene. These crosses were in addition to prior extensive backcrossing of *dilp* mutant strains (Grönke *et al.* 2010).

### Body size

Pupal length and width were determined using an automated detection and measurement system. Segmentation of the pupae for automated analysis was carried out using the “Marker-controlled Watershed” function included in the MorphoJ plugin (Klingenberg 2011) in ImageJ (Schindelin *et al.* 2012; Rueden *et al.* 2017). Briefly, the original image containing the pupae was blurred using the “Gaussian blur” function. A selection of points marking the pupae was then created using the “Find Maxima” function. Next, a new image with the same dimension as the pupae was created, where the individual points were projected onto this original image using the “Draw” function. Then, we labeled each point using the “Connected Components Labeling” function in the MorphoJ plugin (Klingenberg 2011). This image is now the marker image. Upon completion of the marker image, we used the “Morphological Filters” function in the MorphoJ package with the options “operation=Gradient element=Octagon radius=2” to generate a gradient image of the pupae. Using the “Marker-controlled Watershed” function with the gradient image as the input, and the marker image to identify regions of interest outlining the pupae, the width and length of the pupae were obtained by selecting “Fit ellipse” option under the “Set Measurements” menu in ImageJ. Once length and width were determined using this automated measurement system, pupal volume was calculated as previously described (Delanoue *et al.* 2010; Marshall *et al.* 2012; Rideout *et al.* 2012, 2015; Ghosh *et al.* 2014). To measure adult weight, 5-day-old virgin male and female flies were collected and weighed in groups of 10 on an analytical balance.

### Statistical analysis and data presentation

GraphPad Prism (GraphPad Prism version 8.4.2 for Mac OS X) was used to perform all statistical tests and to prepare all graphs in this manuscript. Statistical tests are indicated in figures and figure legends; all *P-*values are listed in Supplementary File S1.

### Data availability

Original images of pupae are available upon request. Raw values for all data collected and displayed in this manuscript are available in Supplementary File S2. The authors affirm that all data necessary for confirming the conclusions of the article are present within the article, figures, tables, and supplementary files. Supplementary material is available at figshare: https://doi.org/10.25387/g3.13191527.

## Results

### Reduced IPC function causes a female-biased decrease in body size

In *Drosophila*, the IPCs located in the brain are an important source of IIS ligands called *Drosophila* insulin-like peptides (Dilps). In larvae, the IPCs synthesize and release Dilp1 (*FBgn0044051*), Dilp2 (*FBgn0036046*), Dilp3 (*FBgn0044050*), and Dilp5 (*FBgn0044048*) into the hemolymph (Brogiolo *et al.* 2001; Ikeya *et al.* 2002; Rulifson *et al.* 2002; Lee *et al.* 2008; Géminard *et al.* 2009). When circulating Dilps bind to the Insulin-like Receptor (InR; *FBgn0283499*) on the surface of target tissues, an intracellular signaling cascade is initiated which ultimately promotes cell, tissue, and organismal size (Chen *et al.* 1996; Böhni *et al.* 1999; Poltilove *et al.* 2000; Britton *et al.* 2002; Werz *et al.* 2009; Almudi *et al.* 2013). The importance of the IPCs in regulating IIS activity and body size is illustrated by the fact that IPC ablation and silencing both reduce IIS activity and decrease overall body size (Rulifson *et al.* 2002; Géminard *et al.* 2009). Yet, the precise requirement for IPCs in body size regulation in each sex remains unclear, as past studies presented data from a mixed-sex population of larvae or reported effects in only a single sex (Rulifson *et al.* 2002; Géminard *et al.* 2009). Because recent studies show that the sex of the IPCs contributes to the sex-specific regulation of body size (Sawala and Gould 2017), we asked how the presence and function of the IPCs affected body size in each sex.

**Figure 1:**
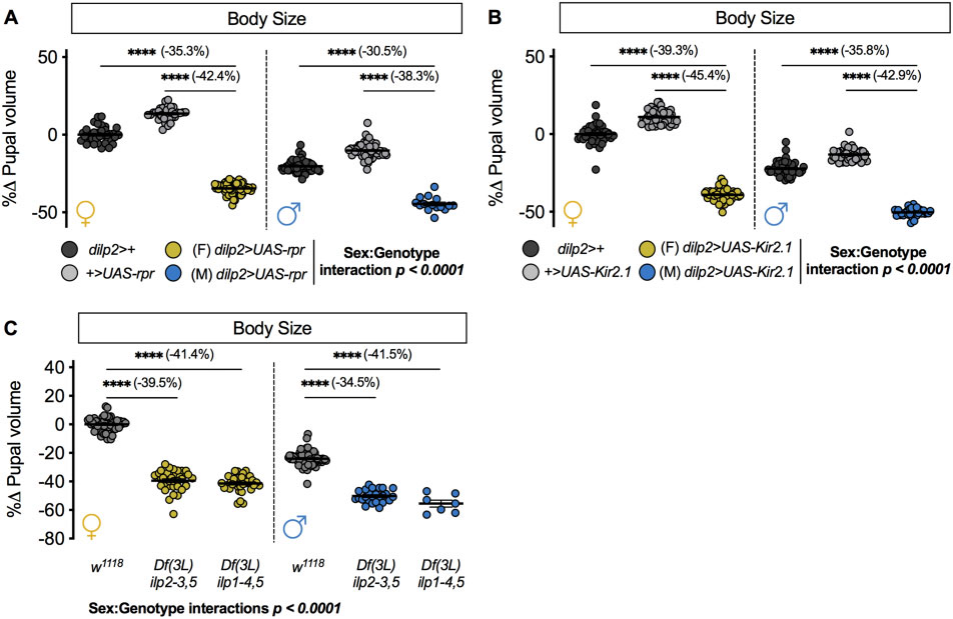
IPC ablation, loss of IPC function, and loss of IPC-derived Dilp ligands all cause a female-biased decrease in growth. (A) Pupal volume in *dilp2>UAS-rpr* females and males compared to *dilp2>+* and *+>UAS-rpr* controls (*P *<* *0.0001 for all comparisons; two-way ANOVA followed by Tukey HSD test). *n *=* *15–71 pupae. (B) Pupal volume in *dilp2>UAS-Kir2.1* females and males compared to both *dilp2>+* and *+>UAS-Kir2.1* controls (*P *<* *0.0001 for all comparisons; two-way ANOVA followed by Tukey HSD test). *n *=* *31–53 pupae. (C) Pupal volume in *Df(3L)ilp2-3,5* and *Df(3L)ilp1-4,5* homozygous females and males compared with sex-matched *w^1118^* controls (*P *<* * 0.0001 for all comparisons; two-way ANOVA followed by Tukey HSD test). *n *=* *7–74 pupae. **** Indicates *P *<* *0.0001; error bars indicate SEM. For all panels, females are shown on the left-hand side of the graph and males are shown on the right-hand side. *P*-values for all sex:genotype interactions are indicated on the graphs.

First, we ablated the IPCs by overexpressing proapoptotic gene *reaper* (*rpr*; *FBgn0011706*) with the IPC-specific GAL4 driver *dilp2-GAL4* (Brogiolo *et al.* 2001; Rulifson *et al.* 2002). This method eliminates the IPCs during development (Rulifson *et al.* 2002). To quantify body size, we measured pupal volume to capture developmental processes such as growth and weight loss that occur during the larval period (Delanoue *et al.* 2010; Testa *et al.* 2013). In females, pupal volume was significantly lower in *dilp2>UAS-rpr* pupae compared with *dilp2>+* and *+>UAS-rpr* control pupae (Figure 1A). In males, pupal volume was also significantly lower in *dilp2>UAS-rpr* pupae compared with control *dilp2>+* and *+>UAS-rpr* pupae (Figure 1A); however, the magnitude of the decrease in body size was greater in females than in males (sex:genotype interaction *P *<* *0.0001; two-way ANOVA). Next, to determine how reduced IPC function affected body size in each sex, we overexpressed the inwardly-rectifying potassium channel *Kir2.1* (Baines *et al.* 2001) using *dilp2-GAL4*. This approach reduces Dilp secretion and lowers IIS activity in a mixed-sex group of larvae (Géminard *et al.* 2009). We found that pupal volume was significantly reduced in *dilp2>UAS-Kir2.1* females compared with *dilp2>+* and *+>UAS-Kir2.1* control females (Figure 1B). In males, pupal volume was reduced in *dilp2>UAS-Kir2.1* pupae compared with *dilp2>+* and *+>UAS-Kir2.1* control pupae (Figure 1B). Because the magnitude of the decrease in female body size was larger than the reduction in male body size (sex:genotype interaction *P *<* *0.0001; two-way ANOVA), this result indicates that inhibiting IPC function caused a female-biased reduction in pupal size. Together, these results identify a previously unrecognized sex-biased body size effect caused by manipulating IPC survival and function. Because previous studies show that IPC loss and IPC inhibition affects several developmental processes that impact final body size, these sex-specific body size effects may be due to sex-specific changes in larval growth, growth duration, and larval weight loss (Okamoto *et al.* 2013; Testa *et al.* 2013; Rideout *et al.* 2015; Sawala and Gould 2017).

### Loss of IPC-derived Dilps causes a female-biased reduction in body size

Given that the larval IPCs produce Dilp1, Dilp2, Dilp3, and Dilp5 (Brogiolo *et al.* 2001; Ikeya *et al.* 2002; Rulifson *et al.* 2002; Lee *et al.* 2008; Géminard *et al.* 2009), we tested whether the loss of some (*Df(3L)ilp2-3,5)*, or all (*Df(3L)ilp1-4,5*), of the IPC-derived Dilps affected pupal size in males and females. While a previous study reported how loss of all IPC-derived *dilp* genes affected adult weight, data from both sexes was not available for all genotypes (Grönke *et al.* 2010). In females, pupal volume was significantly smaller in *Df(3L)ilp2-3,5* pupae, which lack the coding sequences for *dilp2*, *dilp3*, and *dilp5* (Grönke *et al.* 2010), compared with *w^1118^* control pupae (Figure 1C). In males, body size was also significantly reduced in *Df(3L)ilp2-3,5* homozygous pupae compared with *w^1118^* controls (Figure 1C); however, the decrease in body size was significantly greater in females than in males (sex:genotype interaction *P *<* *0.0001; two-way ANOVA). When we measured body size in males and females lacking all IPC-derived Dilps (*Df(3L)ilp1-4,5*), which lack the coding sequences for *dilp1*, *dilp2*, *dilp3*, *dilp4*, and *dilp5* (Grönke *et al.* 2010), we reproduced the female-biased reduction in body size (Figure 1C; sex:genotype interaction *P *<* *0.0001; two-way ANOVA). This reveals a previously unrecognized sex-biased body size effect arising from loss of most, or all, IPC-derived Dilps. Given that several *dilp* genes are known to affect developmental processes that impact body size, these sex-specific body size effects may reflect sex-specific changes in larval growth rate and larval weight loss (Okamoto *et al.* 2013; Testa *et al.* 2013; Rideout *et al.* 2015; Sawala and Gould 2017), and possibly sex-specific effects on the duration of the larval growth period.

### Loss of individual *dilp* genes causes a female-specific decrease in body size

**Figure 2:**
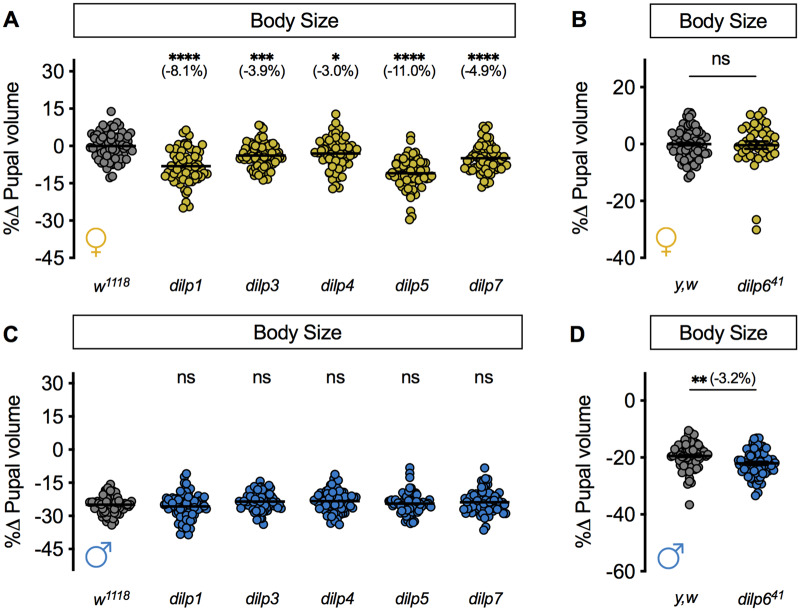
Loss of individual *dilp* genes causes sex-biased effects on growth. (A) In females, pupal volume was significantly reduced compared with *w^1118^* controls in pupae lacking coding sequences for each of the following genes: *dilp1*, *dilp3*, *dilp4*, *dilp5*, and *dilp7* (*P *<* * 0.0001, *P *=* *0.0003, *P *=* *0.0136, *P *<* *0.0001, and *P *<* *0.0001, respectively; one-way ANOVA followed by Dunnett’s multiple comparison test). *n *=* *59–74 pupae. (B) Pupal volume was not significantly different between *y, w* control female pupae and *dilp6^41^* mutant females (*P *=* *0.7634, Student’s *t* test). *n *=* *41–74 pupae. (C) In males, pupal volume was not significantly reduced compared with *w^1118^* controls in pupae lacking coding sequences for each of the following genes: *dilp1*, *dilp3*, *dilp4*, *dilp5*, and *dilp7* (*P *=* *0.7388, *P *=* *0.2779, *P *=* *0.1977, *P *=* *0.9535, and *P *=* * 0.4526, respectively; one-way ANOVA followed by Dunnett’s multiple comparison test). *n *=* *66–79 pupae. (D) Pupal volume was significantly reduced in male *dilp6^41^* pupae compared with *y, w* control males (*P *=* *0.0017, Student’s *t* test). *n *=* *64–70 pupae. * Indicates *P *<* *0.05; ** indicates *P *<* *0.01; *** indicates *P *<* *0.001; **** indicates *P *<* * 0.0001; ns indicates not significant; error bars indicate SEM. Panels A and B display female data; panels C and D show male data.

While Dilp1, Dilp2, Dilp3, and Dilp5 are all produced by the IPCs, previous studies have uncovered significant differences in regulation, secretion, and phenotypic effects of these IPC-derived Dilps (Brogiolo *et al.* 2001; Okamoto *et al.* 2009; Zhang *et al.* 2009; Grönke *et al.* 2010; Cognigni *et al.* 2011; Bai *et al.* 2012; Stafford *et al.* 2012; Linneweber *et al.* 2014; Cong *et al.* 2015; Liu *et al.* 2016; Nässel and Vanden Broeck, 2016; Post *et al.* 2018, 2019; Semaniuk *et al.* 2018; Ugrankar *et al.* 2018; Brown *et al.* 2020). We therefore wanted to determine the individual contributions of IPC-derived Dilps to pupal size in each sex. Furthermore, given that there are non-IPC-derived Dilps that regulate diverse aspects of physiology and behavior (*dilp4*, *FBgn0044049*; *dilp6, FBgn0044047*; and *dilp7, FBgn0044046*) (Grönke *et al.* 2010; Castellanos *et al.* 2013; Garner *et al.* 2018), we wanted to determine the requirement for these additional Dilps in regulating pupal size in each sex. While a previous study measured adult weight as a read-out for body size in *dilp* mutants (Grönke *et al.* 2010), we measured pupal volume to ensure changes to adult weight were not due to altered gonad size (Green and Extavour 2014). We found that pupal volume was significantly smaller in female pupae lacking the coding sequences for *dilp1*, *dilp3*, *dilp4*, *dilp5*, and *dilp7*, respectively, compared with *w^1118^* control females (Figure 2A). These data align well with findings from two recent studies showing a female-specific decrease in larval size caused by loss of *dilp2* (Liao *et al.* 2020; Millington *et al.* 2021). In contrast to most *dilp* mutants; however, there was no significant difference in pupal volume between homozygous *y, w, dilp6*^41^ female pupae and control *y, w* females (Figure 2B). In males, pupal volume was not significantly different between *dilp1*, *dilp3*, *dilp4*, *dilp5*, and *dilp7* mutant pupae and *w^1118^* controls (Figure 2C); however, pupal volume was significantly reduced in *y, w, dilp6^41^* pupae compared with *y, w* controls (Figure 2D). Together, these results extend our current understanding of body size regulation by revealing sex-specific requirements for all individual *dilp* genes in regulating body size. These sex-specific body size effects may be due to a combination of sex-specific effects on larval growth, weight loss in wandering larvae, or growth duration.

### Loss of Dilp binding factor Imp-L2 causes a male-specific increase in body size

**Figure 3:**
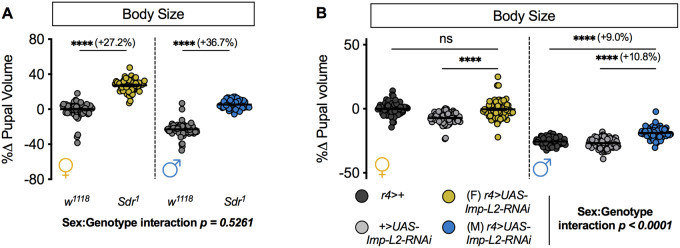
Fat body loss of Dilp binding protein *Imp-L2* has sex-biased effects on growth. (A) Pupal volume in *Sdr^1^* mutant females and males compared with *w^1118^* control females and males (*P *<* *0.0001 for both sexes; two-way ANOVA followed by Tukey HSD test). *n *=* *52–88 pupae. (B) In females, pupal volume was not significantly different between pupae with fat body-specific knockdown of *Imp-L2* (*r4>UAS-Imp-L2-RNAi*) compared with *r4>+* and *+>UAS-Imp-L2-RNAi* control pupae (*P *=* *0.9948 and *P *<* *0.0001, respectively; two-way ANOVA followed by Tukey HSD test), whereas *r4>UAS-Imp-L2-RNAi* males were significantly larger than *r4>+* and *+>UAS-Imp-L2-RNAi* control males (*P *<* *0.0001 for both comparisons; two-way ANOVA followed by Tukey HSD test). *n *=* *70–92 pupae. **** Indicates *P *<* *0.0001; ns indicates not significant; error bars indicate SEM. For all panels, females are shown on the left-hand side of the graph and males are shown on the right-hand side. *P*-values for all sex:genotype interactions are indicated on the graphs.

Once released into the circulation, the Dilps associate with proteins that modulate their growth-promoting effects. For example, Dilp1, Dilp2, Dilp5, and Dilp6 form a high-affinity complex with fat body-derived *ecdysone-inducible gene 2* (*Imp-L2, FBgn0001257*) and Convoluted/*Drosophila* Acid Labile Subunit (Conv/dALS; *FBgn0261269*) (Arquier *et al.* 2008; Honegger *et al.* 2008; Alic *et al.* 2011; Okamoto *et al.* 2013), whereas Dilp3 interacts with Secreted decoy receptor (Sdr) of InR (*FBgn0038279*) (Okamoto *et al.* 2013). Binding of the Imp-L2/dALS complex to individual Dilps likely reduces Dilp binding to InR, as reduced fat body levels of either Imp-L2 or dALS augment IIS activity and increase body size (Arquier *et al.* 2008; Honegger *et al.* 2008; Alic *et al.* 2011). Similarly, loss of Sdr in flies carrying an amorphic *Sdr* allele (*Sdr^1^*), increases IIS activity and increases body size (Okamoto *et al.* 2013). While the Sdr study reported that the magnitude of the increase in adult weight was equivalent in both sexes (Okamoto *et al.* 2013), which we confirm using pupal volume (Figure 3A; sex:genotype interaction *P *=* *0.5261; two-way ANOVA), it remains unclear how the Imp-L2/dALS complex affects pupal size in each sex. Given that one source of secreted Imp-L2 is the fat body (other tissues shown to express Imp-L2 include the corpora cardiaca, insulin-producing cells, and a subset of gut enteroendocrine cells) (Honegger *et al.* 2008; Sarraf-Zadeh *et al.* 2013), we overexpressed an RNAi transgene at equivalent levels in each sex (Millington *et al.* 2021) to reduce *Imp-L2* mRNA levels in the fat body. In females, pupal volume was not significantly different between pupae with fat body-specific overexpression of the *Imp-L2-RNAi* transgene (*r4>UAS-Imp-L2-RNAi*) and control *r4>+* and *+>UAS-Imp-L2-RNAi* pupae (Figure 3B). In contrast, pupal volume was significantly larger in *r4>UAS-Imp-L2-RNAi* male pupae compared with *r4>+* and *+>UAS-Imp-L2-RNAi* control males (Figure 3B). This finding aligns with previous studies showing that *Imp-L2* loss enhances body size (Honegger *et al.* 2008). Furthermore, this finding extends our knowledge by identifying a male-specific effect of reduced fat body *Imp-L2* on pupal size (sex:genotype interaction *P *<* *0.0001; two-way ANOVA), a sex-biased effect that may arise due to sex-specific changes in larval growth, larval weight loss, or developmental timing.

### Altered activity of the intracellular IIS pathway causes sex-biased and non-sex-specific effects on body size

**Figure 4:**
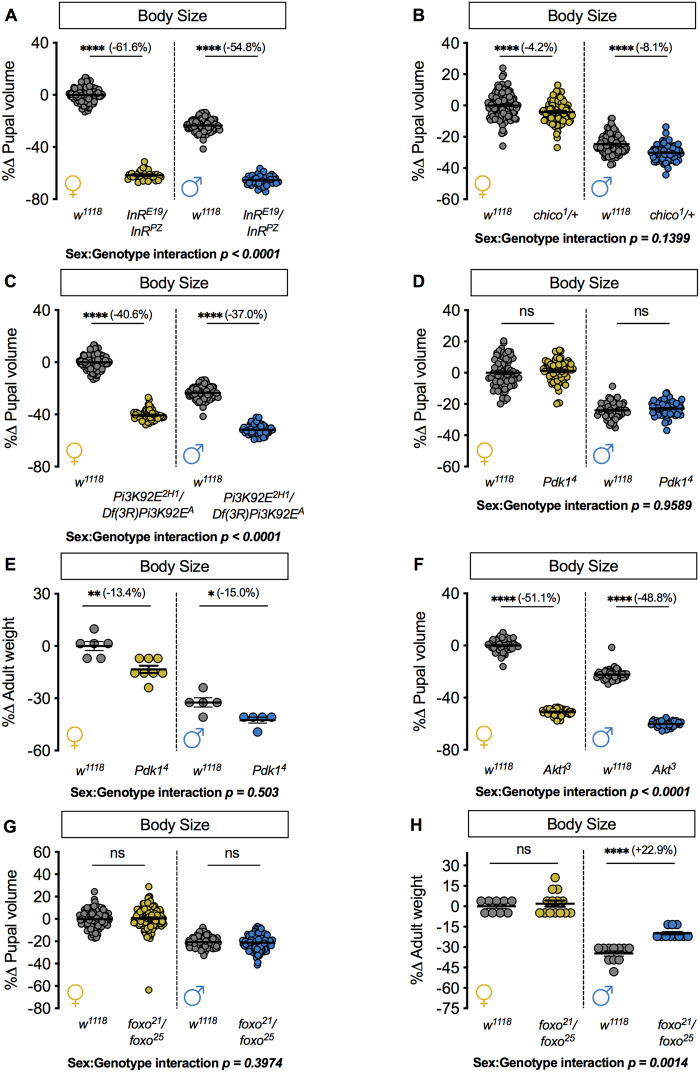
Both sex-biased and non-sex-biased effects on growth arise from loss of intracelllular IIS pathway components. (A) Pupal volume in females and males heterozygous for two hypomorphic *InR* alleles (*InR^E19^/InR^PZ^*) compared with sex-matched *w^1118^* controls (*P *<* *0.0001 for both sexes; two-way ANOVA followed by Tukey HSD test). *n *=* *32–133 pupae. (B) Pupal volume in females and males heterozygous for an amorphic *chico* allele (*chico^1^/+*) compared with sex-matched *w^1118^* controls (*P *<* *0.0001 for both females and males; two-way ANOVA followed by Tukey HSD test). *n *=* *93–133 pupae. (C) Pupal volume in females and males heterozygous for a deficiency and loss-of-function allele of *Pi3K92E (Df(3R)Pi3K92E^A^/Pi3K92E^2H1^*) compared with sex-matched *w^1118^* controls (*P *<* *0.0001 for all comparisons in females and males; two-way ANOVA followed by Tukey HSD test). Note: the *Df(3R)Pi3K92E^A^/Pi3K92E^2H1^* pupae were collected and analyzed in parallel with the *InR^E19^/InR^PZ^* genotype, so the *w^1118^* control genotype data is shared between these experiments. *n *=* *52–133 pupae. (D) Pupal volume was not significant different in either females or males homozygous for a loss-of-function *Pdk1* allele (*Pdk1^4^*) compared with *w^1118^* controls (*P *=* *0.6739 and *P *=* *0.7847, respectively; two-way ANOVA followed by Tukey HSD test). *n *=* *61–84 pupae. (E) Adult weight in *Pdk1^4^* females and males compared with *w^1118^* controls (*P *=* *0.0017 and *P *=* *0.0491 for females and males respectively; two-way ANOVA followed by Tukey HSD test). *n *=* *5–8 biological replicates of ten adult flies. (F) Pupal volume in females and males homozygous for a hypomorphic *Akt1* allele (*Akt1^3^*) compared with sex-matched *w^1118^* controls (*P *<* *0.0001 for both sexes; two-way ANOVA followed by Tukey HSD test). *n *=* *44–60 pupae. (G) In females and males heterozygous for two loss-of-function alleles of foxo (*foxo^21^/foxo^25^*), pupal volume was not significantly different compared with sex-matched *w^1118^* controls (*P *=* *0.8841 and 0.9646, respectively; two-way ANOVA followed by Tukey HSD test). *n *=* *110–153 pupae. (H) In *foxo^21^/foxo^25^* females, adult weight was not significantly different compared with *w^1118^* controls (*P *=* *0.8786; two-way ANOVA followed by Tukey HSD test). In males, adult weight was significantly higher in *foxo^21^/foxo^25^* flies compared with *w^1118^* control flies (*P *<* *0.0001; two-way ANOVA followed by Tukey HSD test). *n *=* *5–8 biological replicates of 10 adult flies. * Indicates *P *<* *0.05; ** indicates *P *<* *0.01; **** indicates *P *<* *0.0001; ns indicates not significant; error bars indicate SEM. For all panels, females are shown on the left-hand side of the graph and males are shown on the right-hand side. *P*-values for all sex:genotype interactions are indicated on the graphs.

In flies, IIS activity is stimulated by Dilp binding to the InR on the surface of target cells (Fernandez *et al.* 1995; Chen *et al.* 1996). This Dilp-InR interaction induces receptor autophosphorylation and recruitment of adapter proteins such as Chico (*FBgn0024248*), the *Drosophila* homolog of mammalian insulin receptor substrate (Böhni *et al.* 1999; Poltilove *et al.* 2000; Werz *et al.* 2009). The recruitment and subsequent activation of the catalytic subunit of *Drosophila* phosphatidylinositol 3-kinase (*Pi3K92E*; *FBgn0015279*) increases the production of phosphatidylinositol (3,4,5)-trisphosphate (PIP_3_) at the plasma membrane (Leevers *et al.* 1996; Britton *et al.* 2002), which activates signaling proteins such as Phosphoinositide-dependent kinase 1 (Pdk1; *FBgn0020386*) and Akt1 (*FBgn0010379*) (Alessi *et al.* 1997). Both Pdk1 and Akt1 phosphorylate many downstream effectors to promote body size (Verdu *et al.* 1999; Cho *et al.* 2001; Rintelen *et al.* 2001). The importance of these intracellular IIS components in regulating organism size is illustrated by studies showing that the loss, or reduced function, of most IIS components significantly decreases body size (Chen *et al.* 1996; Leevers *et al.* 1996; Böhni *et al.* 1999; Weinkove *et al.* 1999; Brogiolo *et al.* 2001; Rulifson *et al.* 2002; Géminard *et al.* 2009; Zhang *et al.* 2009; Grönke *et al.* 2010; Murillo-Maldonado *et al.* 2011). It is important to note that the effects of intracellular IIS components on body size are due to effects on several developmental processes including larval and pupal growth, larval weight loss, and growth duration (Chen *et al.* 1996; Böhni *et al.* 1999; Shingleton *et al.* 2005; Slaidina *et al.* 2009; Grönke *et al.* 2010; Testa *et al.* 2013). Yet, the majority of studies on the regulation of body size by intracellular IIS components were performed in a single- or mixed-sex population of larvae and/or adult flies, and tests for sex-by-genotype interactions were not applied (Fernandez *et al.* 1995; Chen *et al.* 1996; Leevers *et al.* 1996; Böhni *et al.* 1999; Brogiolo *et al.* 2001; Cho *et al.* 2001; Rintelen *et al.* 2001; Ikeya *et al.* 2002; Rulifson *et al.* 2002; Britton *et al.* 2002; Géminard *et al.* 2009; Zhang *et al.* 2009; Grönke *et al.* 2010). Given that recent studies have demonstrated the sex-specific regulation of IIS components such as Akt1 (Rideout *et al.* 2015), we investigated the requirement for each component in regulating pupal size in males and females. In line with previous results showing a female-biased decrease in adult weight in flies heterozygous for two hypomorphic *InR* alleles (Testa *et al.* 2013), we observed a female-biased pupal volume reduction in pupae carrying an additional combination of hypomorphic *InR* alleles (Figure 4A; sex:genotype interaction *P *<* *0.0001; two-way ANOVA) (Fernandez *et al.* 1995; Tatar *et al.* 2001).

To expand these findings beyond *InR*, we measured pupal volume in males and females with whole-body loss of individual intracellular IIS components. Given that we did not obtain viable pupae homozygous for an amorphic allele of *chico* (*chico^1^*), we measured pupal volume in *chico^1^/+* males and females. In *chico^1^/+* females, pupal volume was significantly reduced compared with control *w^1118^* pupae (Figure 4B). In *chico^1^/+* males, pupal volume was reduced compared with control *w^1118^* pupae (Figure 4B). Given that the magnitude of the reduction in pupal volume was similar in males and females (sex:genotype interaction *P *=* *0.1399; two-way ANOVA), reduced *chico* did not cause a sex-biased effect on pupal size. In females heterozygous for one predicted null and one loss-of-function allele of *Pi3K92E*, Df(3R)*Pi3K92E^A^* and *Pi3K92E^2H1^*, respectively (Weinkove *et al.* 1999; Halfar *et al.* 2001), pupal volume was significantly reduced compared with control *w^1118^* pupae (Figure 4C). In Df(3R)*Pi3K92E^A^/Pi3K92E^2H1^* males, we observed a significant reduction in pupal volume (Figure 4C); however, the magnitude of the decrease in pupal size was larger in females compared with males (sex:genotype interaction *P *<* *0.0001; two-way ANOVA). This indicates that loss of Pi3K92E caused a female-biased decrease in body size. Similarly, a previous study showed that heterozygous loss of *Phosphatase and tensin homolog* (*Pten*; *FBgn0026379*), which antagonizes the lipid kinase activity of Pi3K92E to repress growth, also caused a sex-biased increase in pupal volume (Millington *et al.* 2021).

Next, we examined pupal size in males and females homozygous for a loss-of-function allele of *Pdk1* (*Pdk1^4^*). We observed no effect on pupal volume in either sex in *Pdk1^4^* homozygotes (Figure 4D). Given that a previous study showed that adult weight was reduced in *Pdk1^4^*/*Pdk1^5^* (Rintelen *et al.* 2001), we additionally measured adult weight in order to make a direct comparison between our findings and past findings. We found an equivalent body size reduction in *Pdk1^4^* males and females compared with sex-matched control *w^1118^* flies (Figure 4E; sex:genotype interaction *P *=* *0.5030; two-way ANOVA). This suggests that reduced *Pdk1* did not cause a sex-biased reduction in pupal size. One important target of *Pdk1* is the serine/threonine kinase Akt1. In females homozygous for a hypomorphic allele of *Akt1* (*Akt1^3^*), pupal volume was significantly reduced compared with control *w^1118^* pupae (Figure 4F). In *Akt1^3^* males, we observed a significant reduction in pupal size compared with control *w^1118^* pupae (Figure 4F). Given that the magnitude of the decrease in pupal size was larger in females than in males (sex:genotype interaction *P *<* *0.0001; two-way ANOVA), this indicates that loss of Akt1 caused a female-biased effect on pupal size. Together, these findings identify previously unrecognized sex-biased body size effects of reduced *Pi3K92E* and *Akt1*.

One downstream target of IIS that contributes to the regulation of body size is transcription factor *forkhead box, sub-group O* (*foxo*; *FBgn0038197*). When IIS activity is high, Akt1 phosphorylates Foxo to prevent Foxo from translocating to the nucleus (Puig *et al.* 2003). Given that Foxo positively regulates mRNA levels of many genes that are involved in growth repression and catabolism (Zinke *et al.* 2002; Jünger *et al.* 2003; Kramer *et al.* 2003; Alic *et al.* 2011; Slack *et al.* 2011), elevated IIS activity enhances body size in part by inhibiting Foxo (Jünger *et al.* 2003; Kramer *et al.* 2003). Because previous studies show increased Foxo nuclear localization and elevated Foxo target gene expression in males (Rideout *et al.* 2015; Millington *et al.* 2021), we examined how Foxo contributes to pupal size in each sex by measuring body size in females and males heterozygous for two different loss-of-function *foxo* alleles (*foxo^21^/foxo^25^*). In *foxo^21^/foxo^25^* females and males, pupal volume was not significantly different from sex-matched *w^1118^* control pupae (Figure 4G). To directly compare our findings with prior reports on body size effects of *foxo* (Kramer *et al.* 2003; Jünger *et al.* 2003), we also measured adult weight. In adult females, body weight was not significantly different between *foxo^21^/foxo^25^* mutants and control *w^1118^* flies (Figure 4H); however, *foxo^21^/foxo^25^* adult males were significantly heavier than control *w^1118^* males (Figure 4H). Because we observed a male-specific increase in body size (sex:genotype interaction *P *=* *0.0014; two-way ANOVA), our data suggest that Foxo function normally contributes to the reduced adult weight of males. This reveals a previously unrecognized sex-specific role for Foxo in regulating body size. Taken together, these results identify sex-biased effects on pupal size arising from reduced function of some intracellular IIS components (*e.g*., *InR*, *Pi3K92E*, *Akt1*, and *foxo*). In contrast, other intracellular IIS components have non-sex-specific effects on body size (*e.g*., *chico* and *Pdk1*). It will be important in future studies to address how different developmental mechanisms (*e.g*., larval growth, larval weight loss, and growth duration) contribute to both sex-biased and non-sex-biased body size effects of individual IIS components.

## Discussion

An extensive body of work has demonstrated an important role for IIS in promoting cell, tissue, and organismal size in response to nutrient input (Fernandez *et al.* 1995; Chen *et al.* 1996; Böhni *et al.* 1999; Britton *et al.* 2002; Grewal, 2009; Teleman, 2010). More recently, studies suggest that IIS also plays a role in sex-specific body size regulation (Testa *et al.* 2013; Rideout *et al.* 2015; Millington *et al.* 2021). However, potential links between IIS and the sex-specific regulation of body size were inferred from studies using a limited number of genotypes to modulate IIS activity. The goal of our current study was to determine whether the sex-biased body size effects observed in previous studies represent a common feature of genotypes that affect IIS activity. Overall, we found that the loss of most positive regulators of IIS activity caused a female-biased reduction in body size. On the other hand, loss of genes that normally repress IIS activity caused a male-specific increase in body size. Thus, most changes to IIS activity cause sex-biased, or sex-specific, effects on body size (summarized in Table 1), highlighting the importance of collecting and analyzing data from both sexes separately in studies that manipulate IIS activity and/or examine IIS-responsive phenotypes (*e.g*., lifespan and immunity).

**Table 1:** Summary of sex-biased effects of IIS pathway manipulations on body size

	Genetic manipulation	Female-biased	Male-biased	Non-sex-specific	Percent change body size
**Reduced circulating Dilps**	IPC ablation	Yes	—	—	F: –34.5% M: –30.5%
	IPC silencing	Yes	—	—	F: –39.3% M: –35.8%
	*dilp2-3,5*	Yes	—	—	F: –39.5% M: –34.5%
	*dilp1-4,5*	Yes	—	—	F: –41.4% M: –41.5%
	*dilp1*	Yes	—	—	F: –8.1% M: ns
	*dilp3*	Yes	—	—	F: –3.9% M: ns
	*dilp4*	Yes	—	—	F: –3.0% M: ns
	*dilp5*	Yes	—	—	F: –11.0% M: ns
	*dilp6*	—	Yes	—	F: ns M: –3.2%
	*dilp7*	Yes	—	—	F: –4.9% M: ns
**Increased circulating Dilps**	*Sdr*	—	—	Yes	F: –27.2% M: ns
	*Fat body Imp-L2*	—	Yes	—	F: ns M: +9.0%
**Intracellular IIS pathway**	*InR*	Yes	—	—	F: –61.6% M: –54.8%
	*chico^1^/+*	—	—	Yes	F: –4.2% M: –8.1%
	*Pi3K92E*	Yes	—	—	F: –40.6% M: –37.0%
	*Pdk1*	—	—	Yes	F: –13.4% M: –15.0%
	*Akt1*	Yes	—	—	F: –51.1% M: –48.8%
	*Foxo*	—	Yes	—	F: ns M: +22.9%

All data used in this summary table are derived from pupal volume experiments, except for *Pdk1* and *foxo*, where adult weight is shown.

One important outcome from our study was to provide additional genetic support for IIS as an important regulator of the sex difference in body size. Data implicating IIS in the sex-specific regulation of body size first emerged from a detailed examination of the larval stage of development in wild-type flies of both sexes (Testa *et al.* 2013). In this study, the authors reported a female-biased body size reduction in flies with decreased InR function (Testa *et al.* 2013). A subsequent study extended this finding by uncovering a sex difference in IIS activity: late third-instar female larvae had higher IIS activity than age-matched males (Rideout *et al.* 2015). The reasons for this increased IIS activity remain incompletely understood; however, Dilp2 secretion from the IPCs was higher in female larvae than in males (Rideout *et al.* 2015). Given that Dilp2 overexpression is known to augment IIS activity and enhance body size (Ikeya *et al.* 2002; Géminard *et al.* 2009), these findings suggest a model in which high levels of circulating Dilp2 (and possibly other Dilps) are required in females to achieve and maintain increased IIS activity and a larger body size in nutrient-rich conditions. In males, lower circulating levels of Dilp2 lead to reduced IIS activity and a smaller body size. If this model is accurate, we predict that female body size will be more sensitive to genetic manipulations that reduce Dilp ligands and/or IIS activity. Previous studies provided early support for this model by demonstrating a female-biased reduction in body size due to strong *InR* inhibition and *dilp2* loss (Testa *et al.* 2013; Liao *et al.* 2020; Millington *et al.* 2021). Now, we provide strong genetic support for this model using multiple genetic manipulations to reduce IIS activity, confirming that *Drosophila* females depend on high levels of IIS activity to promote increased body size. One potential reason for this high level of IIS activity in females is to ensure successful reproduction, as IIS activity in females regulates germline stem cell divisions, ovariole number, and egg production (LaFever and Drummond-Barbosa 2005; Hsu *et al.* 2008; Hsu and Drummond-Barbosa 2009; Grönke *et al.* 2010; Green and Extavour 2014). Unfortunately, this elevated level of IIS activity shortens lifespan, revealing an important IIS-mediated tradeoff between fecundity and lifespan in females (Broughton *et al.* 2005).

A second prediction of this model is that augmenting either circulating Dilp levels or IIS activity will enhance male body size. Indeed, we demonstrate that loss of *Imp-L2*, which increases free circulating Dilp levels (Arquier *et al.* 2008; Honegger *et al.* 2008; Alic *et al.* 2011; Okamoto *et al.* 2013), and loss of *foxo*, which mediates growth repression associated with low IIS activity (Jünger *et al.* 2003; Kramer *et al.* 2003), both cause a male-specific increase in body size. Together, these findings suggest that the smaller body size of male pupae is partly due to low IIS activity. While the reason for lower IIS activity in males remains unclear, studies show that altered IIS activity in either of the two main cell types within the testis compromises male fertility (Ueishi *et al.* 2009; McLeod *et al.* 2010; Amoyel *et al.* 2014, 2016). Future studies will therefore need to determine how males and females each maintain IIS activity within the range that maximizes fertility. In addition, it will be important to determine whether the female-biased phenotypic effects of lower IIS activity that we observe, and which are prevalent in aging and lifespan studies (Clancy *et al.* 2001; Holzenberger *et al.* 2003; Magwere *et al.* 2004; Van Heemst *et al.* 2005; Selman *et al.* 2008; Regan *et al.* 2016; Kane *et al.* 2018) extend to additional IIS-associated phenotypes (*e.g*., immunity and sleep) (DiAngelo *et al.* 2009; Cong *et al.* 2015; Roth *et al.* 2018; Suzawa *et al.* 2019; Brown *et al.* 2020).

Another important task for future studies will be to gain deeper insight into sex differences in IPC function, as one study identified sex-specific Dilp2 secretion from the IPCs (Rideout *et al.* 2015). Indeed, recent studies have revealed the sex-specific regulation of one factor (*stunted*, *FBgn0014391*) that influences Dilp secretion from the IPCs (Delanoue *et al.* 2016; Millington *et al.* 2021), and female-specific phenotypic effects of another factor that influences IPC-derived Dilp expression (Woodling *et al.* 2020). Together, these studies suggest that sex differences in IPC function and circulating Dilp levels exist, and may arise from the combined effects of multiple regulatory mechanisms. Given that our knowledge of IPC function has recently expanded in a series of exciting studies (Meschi *et al.* 2019; Oh *et al.* 2019), more work will be needed to test whether these newly discovered modes of IPC regulation operate in both sexes. Furthermore, it will be important to ascertain how sex differences in the IPCs are specified. One recent study showed that *Sex-lethal* (*Sxl*; *FBgn0264270*), a key regulator of female sexual development, acts in the IPCs to regulate the male–female difference in body size (Sawala and Gould 2017). By studying how *Sxl* function alters IPC gene expression, activity, and connectivity, it will be possible to gain mechanistic insight into the sex-specific regulation of body size.

Beyond an improved understanding of sex differences in IPC function, it will be essential to study the sex-specific regulation of *dilp* genes and Dilp proteins, as we show female-specific effects on body size in pupae lacking most individual *dilp* genes. While two previous studies report female-biased effects of loss of *dilp2* (Liao *et al.* 2020; Millington *et al.* 2021), this is the first report of a female-specific role for *dilp1*, *dilp3*, *dilp4, dilp5*, and *dilp7* in promoting growth. While the female-specific effect of *dilp2* loss on pupal size aligns with the fact that female larvae have higher circulating Dilp2 levels (Rideout *et al.* 2015), much remains to be discovered about the sex-specific regulation of most *dilp* genes and Dilp proteins. For example, females have an increased number of *dilp7*-positive cells compared with males (Castellanos *et al.* 2013; Garner *et al.* 2018); however, it is unclear whether these additional *dilp7*-positive cells in females augment circulating Dilp7 levels. A full understanding of the female-specific effects that accompany loss of most individual *dilp* genes will therefore require more knowledge of sex differences in the regulation of *dilp* genes and Dilp proteins. In addition to revealing the female-specific effects of many *dilp* genes on pupal size, we are also the first to report a male-specific body size effect of *dilp6*. Normally, Dilp6 function sustains growth in nonfeeding conditions, and is upregulated in low-nutrient contexts (Slaidina *et al.* 2009). Interestingly, male larvae have lower IIS activity than age-matched females (Rideout *et al.* 2015), where decreased IIS activity phenocopies a low-nutrient environment (Britton *et al.* 2002). Therefore, one potential explanation for the male-specific effect of *dilp6* loss on pupal size is that reduced IIS activity in normal males leads to an increased reliance on Dilp6 to maintain body size. In females, higher levels of potent growth-promoting Dilp2 (Ikeya *et al.* 2002), and possibly other Dilps, promote IIS activity to minimize the requirement for Dilp6 function. This possibility will be important to test in future studies, alongside experiments to address a potential sex-specific role for other regulators of *dilp6*/Dilp6 including steroid hormone ecdysone and the Toll signaling pathway (Slaidina *et al.* 2009; Suzawa *et al.* 2019). Furthermore, as our knowledge of how individual *dilp* genes affect larval development and physiology continues to grow, analyzing data from both sexes will play an important role in extending knowledge of the mechanisms underlying sex differences in body size and other IIS-associated traits.

In contrast to the female-biased effects of most genetic manipulations that reduced Dilp availability, we observed both sex-biased and non-sex-biased effects on body size in pupae with reduced function of key intracellular IIS components. For example, reduced InR, Pi3K92E, and Akt1 function caused a female-biased reduction in body size, whereas there was an equivalent reduction in male and female body size due to lower *chico* and *Pdk1* function. While more information on larval growth, developmental timing, and larval weight loss are needed to fully understand why different IIS components have sex-biased or non-sex-biased body size effects, one recent study showed that heterozygous loss of *chico* caused insulin hypersecretion (Sanaki *et al.* 2020). Given that hyperinsulinaemia contributes to insulin resistance, and that insulin resistance decreases *Drosophila* body size (Musselman *et al.* 2011; 2017; Pasco and Léopold 2012), more studies will be needed to determine whether the smaller body size of *chico^1^/+* male and female pupae, and possibly *Pdk1* mutant flies, can be attributed to insulin resistance. In fact, more knowledge of sex-specific tissue responses to insulin is urgently needed in male and female flies, as studies in mice and humans have identified sex differences in insulin sensitivity (Geer and Shen 2009; Macotela *et al.* 2009). Because *Drosophila* is an emerging model to understand the mechanisms underlying the development of insulin resistance (Musselman *et al.* 2011), this knowledge would help determine whether flies are a good model to investigate the sex-biased incidence of diseases associated with insulin resistance, such as the metabolic syndrome and type 2 diabetes (Mauvais-Jarvis 2015).
